# First case report of dermatitis associated with *Leporacarus gibbus* in cat

**DOI:** 10.1186/s12917-020-02681-0

**Published:** 2021-01-06

**Authors:** Mirabela Oana Dumitrache, Adriana Györke, Gianluca D’Amico, Viorica Mircean

**Affiliations:** Department of Parasitology and Parasitic Diseases, University of Agricultural Sciences and Veterinary Medicine Cluj-Napoca, Calea Mănăştur 3-5, Cluj-Napoca 400372, Cluj, Romania

**Keywords:** *Leporacarus gibbus*, Cat, Dermatitis

## Abstract

**Background:**

*Leporacarus gibbus* is a highly specific acarian parasitizing in rabbits, with a proven zoonotic potential. While the majority of cases of *L. gibbus* infestation are asymptomatic, several cases of pruritic cutaneous condition in both laboratory and pet rabbits were reported. Up to date, *L. gibbus* has not been linked with clinical signs in any other species than rabbits and humans.

**Case presentation:**

This case report described the clinical case of a 14-month-old cat with a dermatitis linked to *L. gibbus*. Mites specimens were collected by brushing, followed by light microscopy examination and species identification. To the best of our knowledge, this is the first report of *L. gibbus*-related dermatitis in cat.

**Conclusions:**

*L. gibbus *infestation should be considered as a possible differential diagnosis of pruritic skin conditions in cat.

## Background

*Leporacarus gibbus* (formerly *Listrophorus gibbus*), the rabbit fur mite, belongs to the family Listrophoridae, division Psoroptida, order Astigmata [1]. The life cycle of the parasite is characterized by a complete metamorphosis and occurs entirely on the rabbit coat. *L. gibbus* feeds on sebum and skin epithelial cells. Different studies questioning its pathogenicity. Due to the asymptomatic evolution of the majority of cases, *L. gibbus* is also considered as commensal, which can be commonly found on the rabbits’ skin [2, 3]. Despite of being considered as a cosmopolitan species for both wild and domestic rabbits in Europe, only few studies are available on this topic. There is a lack of epidemiological studies and reports of *L. gibbus* in many countries. In addition, the description of the life cycle and contamination method remained unclear for many years [3]. It is though that *L. gibbus* is generally well tolerated by the rabbit host, but a hypersensitivity reaction could determine the occurrence of clinical signs [4]. *L. gibbus* has been occasionally reported as the aetiological agent of a pruritic cutaneous condition in both laboratory and pet rabbits. Clinically, this ectoparasitosis is characterized by pruritus, moist dermatitis, poorly demarcate alopecia, erythema and scaling on the dorsum and hindlimbs [3, 5]. Although it is considered as uncommon in humans, several case reports confirmed its zoonotic potential [5]. Up to date, *L. gibbus* has not been linked with clinical signs in any other species than rabbits and humans [1, 5].

## Case presentation

A 14-month-old, sterilised female Ragdoll cat, only pet, living indoor, was presented with a history of one year of pruritus. The anamnesis revealed that the cat was purchased online a year ago and the owner observed the presence of generalised pruritus, the lack of hair on the tail and face, and black cerumen in the both ears during the first few days following acquisition. At that time, the patient was diagnosed by a primary care veterinarian with otodectic mange. The treatment applied was a single spot-on application of 45 mg selamectin (Stronghold ®, Zoetis) that lead to clinical resolution of the otodectic mange and skin lesions, and a substantial improvement of the generalised pruritus. The later symptom persisted at a low level of intensity during the following months. Furthermore, transitory digestive signs (vomiting, diarrhoea) occurred. At the age of 13 months, an intensification of the pruritus was observed. The owner linked this event with the antirabic vaccination. The same practitioner recommended an exclusion diet with an anallergenic food and two doses of dexamethasone (Dexamethasone ®, Kepro) administrated at 3 days interval. After a month of therapy, the case was referred to our clinic because only a slightly improvement of the symptoms has been noticed.

**Fig. 1:**
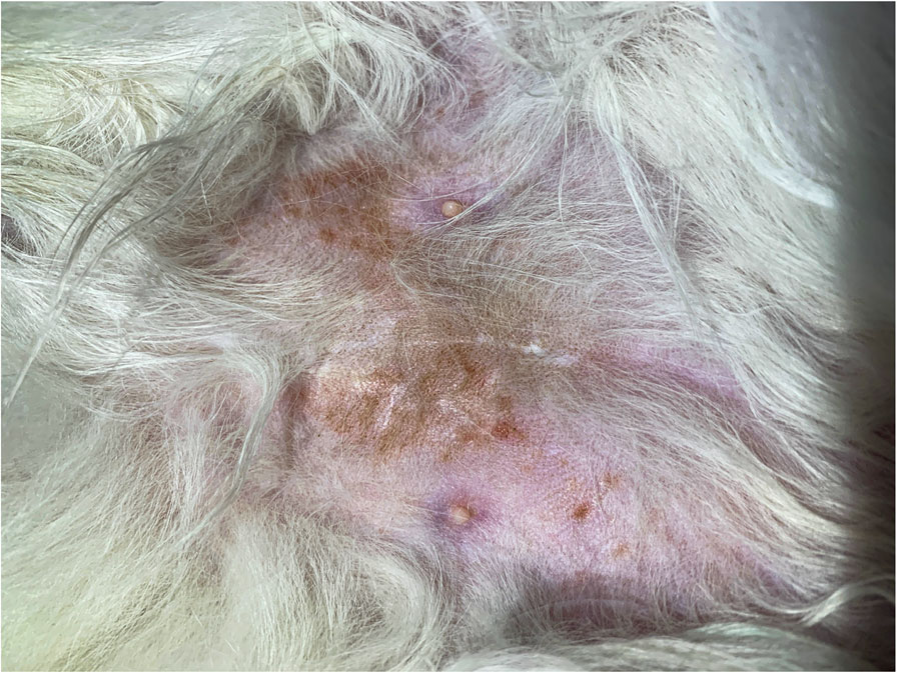
Ventral aspect of a cat infested with *L. gibbus*

**Fig. 2:**
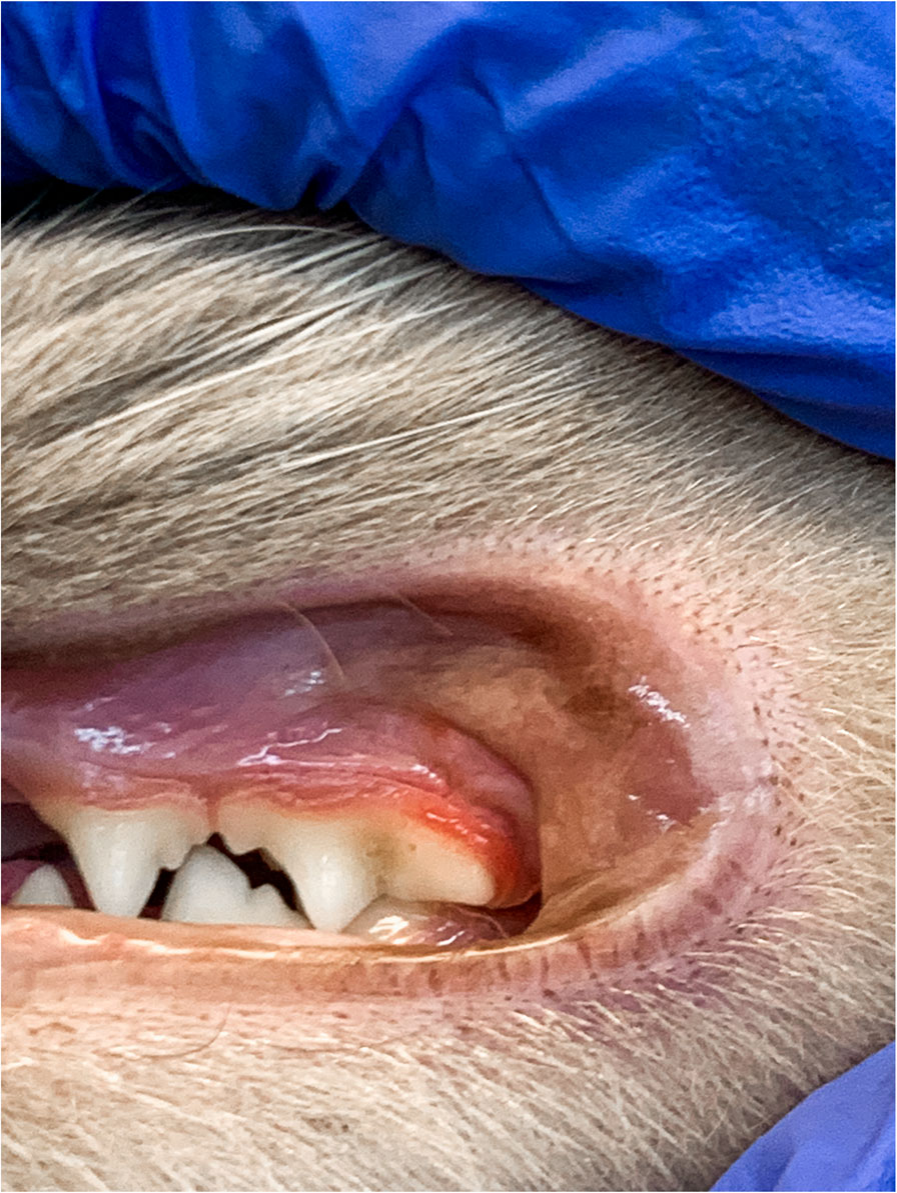
Gingivitis associated with *L. gibbus* infestation in a cat

At the moment of presentation, the clinical examination revealed a self-induced alopecia on the abdomen, the presence of papulosquamous lesions in the same region (Fig. 1), and gingivitis (Fig. 2).

**Fig. 3:**
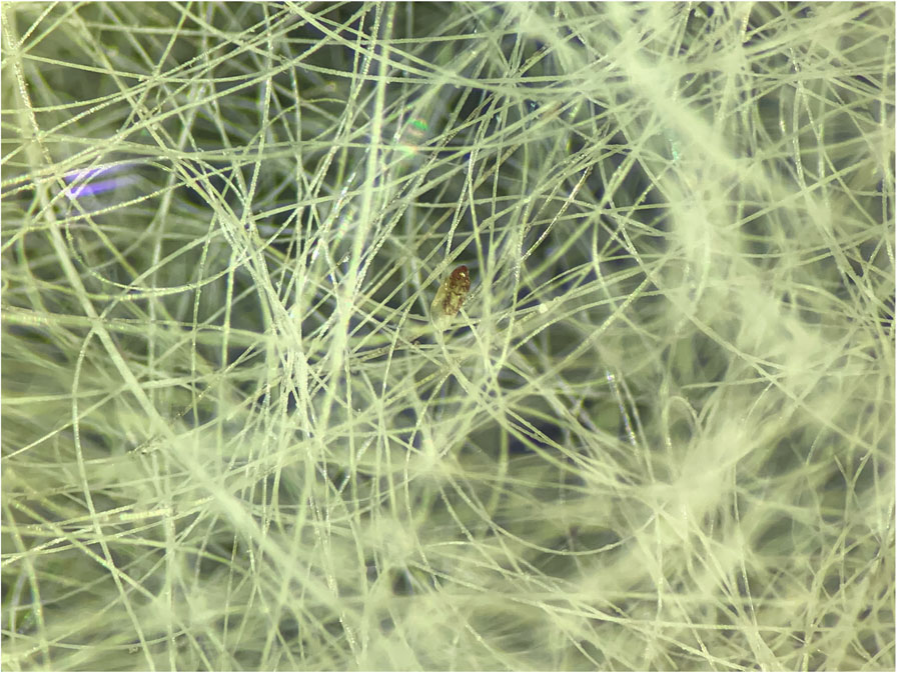
Stereomicroscope examination of the coat brushing

**Fig. 4:**
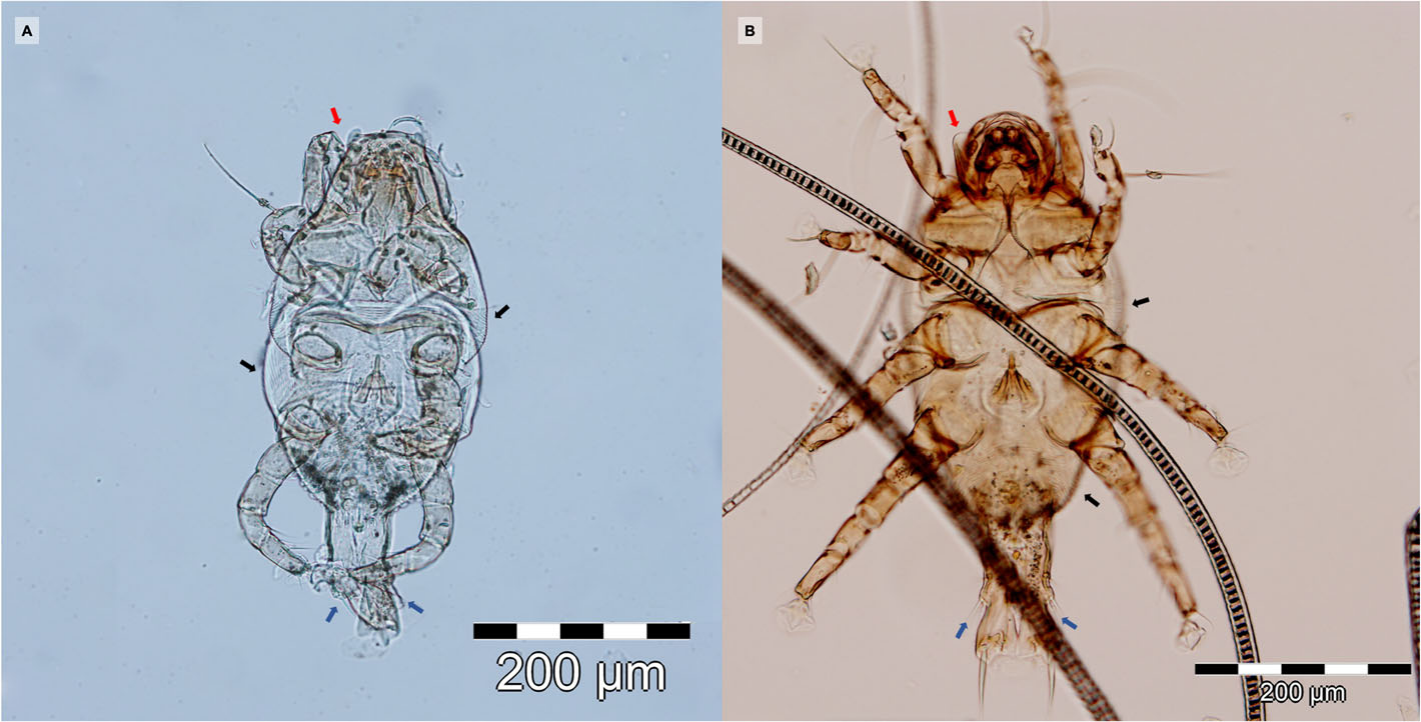
Microscopic image of *L. gibbus* male, from cat (**a**) and from rabbit (**b**), showing finely striated cuticle (black arrows), sclerotised “dark hooded” gnathostoma (red arrow), two elongated adanal processes (blue arrows)

Both skin scrapings and scotch test proved negative. However, stereomicroscope examination of the coat brushing revealed the presence of several mites. The image of a female mite at a low power objective (3x) is provided (Fig. 3). The mite specimens were collected and the microscopic examination using the 10x objective confirmed the presence of *L. gibbus* based on the following morphological characteristics: subcylindrical body, finely striated cuticle, sclerotised gnathostoma, two striated membranous flaps at each of the coxae of the first pair of legs, and presence of two elongated adanal processes and distinct adanal suckers in males (Fig. 4a) [3]. No other mite species were visualized. For comparison, a *L. gibbus* male collected from a rabbit is provided (Fig. 4b). In order to confirm the morphological species identification, the genomic DNA was extracted from specimens collected from the cat (*n* = 2) and from the rabbit (*n* = 10), by using a commercial kit (Isolate II Genomic DNA Kit, Bioline, London, UK) following the manufacturer’s instructions. The COI gene fragment was PCR-amplified using primer pairs bcdF05/bcdR04 [6, 7]. The PCR products were purified using FavorPrep GEL/PCR Purification Mini Kit (Favorgen Biotech Corp., Taiwan) and further sequenced (Macrogen Europe). The obtained sequences were compared with the available sequence in GenBank (acc. no. GQ864335.1). The BLAST analysis showed 92.39%, and 92.21% similar degree identity with *L. gibbus* for the cat, and rabbit strains respectively. The sequences obtained in this study from the parasites collected from the cat and from rabbits were submitted to the GenBank database under the following accession numbers: MW286180 and MW286214, respectively.

To evaluate one of the most common causes of immunosuppression in cats [8], a rapid test for FIV/FeLV infections (SNAP FIV/FeLV Combo Test, IDEXX) was carried out, but tested negative. The cat was treated with two spot-on applications of 45 mg selamectin (Stronghold ®, Zoetis) at two weeks interval, a protocol proved to be effective by Birke et al. [1]. Coat brushing tested negative for mites at 7 days after the second dose of selamectin. The skin lesions and the pruritus disappeared within the first 14 days of treatment. The owner did not show any lesions on the course of the disease evolution nor at the time of rabbit examination.

## Discussions and conclusion

Although pruritus is a frequent sign of dermatological conditions in cats, this species develops less relevant diagnostic lesions than those observed in dogs. Moreover, the primary inflammatory lesions that may be present and could be relevant for identification of the pruritus cause are frequently transformed in secondary lesions due to the different behaviours triggered by itch (e.g. scratching, licking) [9]. Given this, the diagnosis approach of a pruritic skin condition should be made according to the cutaneous reaction patterns [9]. The causes of pruritus in cats are various, such as hypersensitivity dermatitis, ectoparasites, fungal infections, bacterial infections, or cutaneous reaction to systemic diseases [9, 10]. As diverse as the causes are, as various the clinical patterns are: eosinophilic syndrome, miliary dermatitis, head and/or neck excoriation, regional or generalized scaling and/or crusting dermatoses, and symmetrical self-induced alopecia [10]. Nevertheless, for the development of an efficient treatment approach of feline pruritus, the case history might be of a great importance.

In this case report, the history of the cat, its origin, the young age, the lack of anti-parasitic treatments, and the clinical presentation with the self-induced alopecia clinical pattern suggested a parasitic disease as first hypothetical diagnosis, followed by hypersensitivity dermatitis that could have been caused by flea bite, cutaneous adverse food reactions, and/or atopic dermatitis [10]. Cats with nonseasonal pruritus and suggestive clinical patterns should undergo a dietary elimination-challenge trial to determine the importance of food allergen in the aetiology of a condition [10]. A restriction diet of 6 to 8 weeks is recommended to be followed. In our case, the cat was already following a strict diet with anallergenic food at the time of the initial examination. The lack in clinical signs improvement following the exclusion diet and anti-pruritic treatment with corticosteroids allowed us to eliminate food allergy as a hypothetical diagnosis. It is well known that in cats with food allergy, pruritus, which is the main complain of owners, is corticoid-resistant in the majority of cases [11]. Altogether, the parasitic aetiology remained a possible cause and additional diagnostic test were performed to confirm or to exclude fleas’ infestation, cheyletiellosis, demodicosis, pediculosis and/or *Lynxacarus radovskyi* infestation.

While skin scrapings and scotch test yielded negative results, the stereomicroscope examination of the coat brushing confirmed our hypothesis of a parasitic skin condition by revealing the mites. Initially, we suspected an infestation with *L. radovskyi*, due to the similar morphology of the mites and classification in the same family [12]. The presence of some uncharacteristic morphological features, as well as the few cases reported in the scientific literature [13] and *L. radovskyi* distribution in tropical regions [14] leaded us to perform a more detailed examination. Surprisingly, the microscopic identification based on the morphological characteristics revealed *L. gibbus* as the aetiological agent. Given that the cat was living exclusively indoor and considering its origin, we can assume that the contamination occurred through the close contact with infested rabbits from the pet shop. Diagnosis of *L. gibbus* in rabbits is challenging because the tape test could be inefficient due to the localization of the parasite on the first third of the hair shaft. This seems to be the principal cause of underdiagnosis in rabbits [1] and might explain why the scotch test we performed was negative in cat.

*L. gibbus* has a zoonotic potential and it has been linked twice with papular eruptions in humans [5, 15]. Both reports highlight the difficulty in demonstrating the aetiology of the lesions in rabbit’s owners because no mites were detected on their skin. In one report, the owners’ dermatologist admitted a possible parasitic aetiology and suggested that the pruritic erythematous papules were most likely the result of a hypersensitivity reaction to mites’ biting [5]. Morover, the resolution of the owners’ skin lesions following the specific treatment of rabbits is an indirect, but substantial proof for that incriminates *L. gibbus* [5]. Despite of being considered as a common parasite of rabbits or even a commensal of this species, only few reports on the zoonotic character of *L. gibbus* are available. Hence, we can suspect that that the zoonotic transmission is linked to a certain immunologic status of the owner, like in the case of other mites [16], or uncommon.

Several treatment methods against *L. gibbus* have been proposed [1, 2, 17]. Although both imidocloprid plus permethrin and selamectin proved to be effective against this parasite, selamectin seems to eliminate the infestation more rapidly and is considered that a single selamectin application has a 100% efficacy against *L. gibbus*. However, some authors recommend monthly treatment until no live mites are detected [16]. These reports could explain the results obtained in our case. The first spot-on treatment most likely has eliminated most of the parasites and allowed a temporary improvement of cat’s condition. As no other treatment followed this first administration, the few parasites remaining on the skin continued their lifecycle and determined a low intensity but permanent pruritus during the following months. The exacerbation of pruritus following vaccination could be related to the stress associated with this procedure.

Infestation with other mites such as *L. radovskyi* in cats, were previously associated with gastrointestinal signs, gingivitis, fever, vulvitis/proctitis, weight loss, etc. [12]. However, as now other similar cases are reported, it is difficult to correlate the presence of the non-dermatological signs identified in our patient, with *L. gibbus* infestation. However,

The findings of this case report suggest that *L. gibbus* infestation, although uncommon, should be considered as a possible differential diagnosis of pruritic skin conditions in cat.AbbreviationPCR: polymerase chain reactions; FIV: feline immunodeficiency virus; FeLV: feline leukemia virus.

## Data Availability

All relevant data are within this paper.
